# Depressive symptoms and anxiety among women with a history of abortion living in urban slums of Bangladesh

**DOI:** 10.1186/s40359-023-01224-0

**Published:** 2023-07-04

**Authors:** Kamrun Nahar Koly, Jobaida Saba, Md Arif Billah, Alba McGirr, Tithi Sarker, Mahbubul Haque, Elvina Mustary, S. M. Manzoor Ahmed Hanifi, Farzana Begum

**Affiliations:** 1 Health System and Population Studies Division, International Centre for Diarrhoeal Disease Research, Bangladesh (icddr,b), Dhaka 1212 Mohakhali, Bangladesh; 2 Reproductive Health Services Training and Education Program (RHSTEP), Mirpur, 1216 Dhaka Bangladesh

**Keywords:** Spontaneous abortion, Women, Depression, Anxiety, Urban slum, Bangladesh

## Abstract

**Background:**

Globally, major emphasis has been placed on understanding the physiological consequences of losing a pregnancy. However, its mental health impact on socially disadvantaged women remains unexplored. To further inform the field the present study investigated the prevalence and factors associated with the development of depressive symptoms and anxiety among women with a history of spontaneous abortion living in the urban slums of Dhaka, Bangladesh.

**Methods:**

Information was obtained from 240 women who experienced a spontaneous abortion from July 2020 to December 2021. It was obtained through the urban health and demographic surveillance system (UHDSS) survey. Generalized Anxiety Disorder (GAD-7) and Patient Health Questionnaire (PHQ-9) were used to measure mental health symptoms. Bivariate and multivariate linear regression analyses were performed to assess the associated factors with the mental health outcomes.

**Results:**

Of the 240 women, majority (77.50%) of the women experienced mild to severe depressive symptoms and more than half (58.75%) of the respondants experienced mild to severe anxiety, within one and half years of experiencing spontaneous abortion. A higher level of education and being employed were protective factors for anxiety and depressive symptoms, respectively. However, women with higher sexual and reproductive health rights (SRHR) knowledge had significantly increased anxiety and depressive symptoms. In contrast, receiving post-abortion care (PAC) was associated with decreased anxiety and depressive symptoms.

**Conclusion:**

The findings indicate that ensuring access to affordable PAC services and integrating mental health services into the standard PAC service package is crucial. This study also emphasizes the importance of providing education for women living in urban slums and encouraging them to participate in economic activities.

**Supplementary Information:**

The online version contains supplementary material available at 10.1186/s40359-023-01224-0.

## Background

Spontaneous abortion, also called, a miscarriage, is considered as poor Sexual and Reproductive Health (SRH) outcome with higher consequences [1]. Around 23 million spontaneous abortions occur annually, equating to 44 pregnancy losses every minute [2]. Women at higher risk of pregnancy loss include those under the age of 20 or over 35 years, particularly those who experienced intimate partner violence, have a very low or very high body-mass index, have a history of miscarriages, smoking, stress, or are exposed to pesticides and pollution [3]. These issues are common among people of lower and middle-income countries (LMICs) especially urban slum dwellers, due to inadequate understanding and access to sexual and reproductive health rights (SRHR)-related information and services [4–7]. Spontaneous abortions have severe psychological impacts partly because they are unexpected by nature and have multifaceted impacts [8].

Depression and anxiety are the most common mental health conditions among women from both LMICs and high-income countries who experience spontaneous abortion [9]. In Mexico, 41.70% of women who experienced spontaneous abortions had depression and anxiety [10]. A large scale case-control study conducted in China also reported anxiety and depressive symptoms among women with a history of spontaneous abortions, and the risk was higher among those who had recurrent miscarriages [11]. Another Chinese longitudinal study reported that 55% of women stated depressive symptoms (≥ 4 on the General Health Questionnaire) three months after the spontaneous abortion [12]. This was also found to be the case in the United Kingdom, where a similar group of women exhibited moderate to severe anxiety and depressive symptoms [13]. A systematic review reported the major risk factors for depression and anxiety among women with spontaneous abortions [14]. A review stated anxiety was common among women with a history of poor mental health, lower spousal support, no children, a history of miscarriage, and unplanned and assisted pregnancy. Along with these factors, depression was also found among women of younger age, poor relationships with partners and a history of infertility [9]. Despite the aforementioned burden of depression and anxiety among women experiencing spontaneous abortion reported worldwide, no previous evidence has been found in Bangladesh that interlinked these mental health issues among women with spontaneous abortion, especially those living in urban slums.

In Bangladesh, about 7 million people (~ 42%) in urban areas live in slums. They are especially at risk of poor health and are deprived of basic health care and family planning services [15, 16]. Female slum-dwellers, often face socio-economic challenges, i.e., poverty, low education, violence, food security, and other social disadvantages that exacerbate mental health impacts [17–23]. Research is lacking in Bangladesh into these factors, which together with the inequitable distribution of major health services, could put additional strain on the mental health of the slum women. Moreover, early marriages, low prevalences of family planning, and early & unwanted pregnancies are common in urban slum women due to inadequate access to health and SRH services [15, 24]. Consequently, most early and unintended pregnancies can potentially result in spontaneous abortion. However, the uptake of SRHR and post-abortion care (PAC) services are notably low in the slums due to high costs and a lack of awareness [25–29]. Additionally, restrictive laws and costly formal care push vulnerable women to perform unsafe abortions. They then fail to use the post-abortion care (PAC) services [4, 30, 31].

The available PAC service packages accessible to Bangladeshi slum women comprise emergency treatment for complications and advice regarding family planning [32], but there is no arrangement for any services related to mental healthcare. However, global studies indicated specific mental health support interventions, such as counseling, are beneficial for women after a spontaneous abortion. [33–36]. Moreover, previous studies stressed the need for integrating psychosocial support services into the PAC package for women who experienced pregnancy loss or abortion [9, 34].

The mental health consequences of spontaneous abortion among slum-dwelling women are largely understudied. Also, very few studies have explored the association of factors such as employment, education, wealth index, SRHR knowledge, and utilization of PAC services with the mental health of these women. Therefore, it is essential to explore the mental health issues of women who have experienced spontaneous abortion in poor-resource settings and informal settlements. Furthermore, such evidence is empirical and can guide the relevant policymakers to integrate mental healthcare into PAC services in the future. This study was conducted to estimate the prevalence and possible predictors of anxiety and depression in women with experience of spontaneous abortion living in slum areas in Dhaka, Bangladesh.

## Methods

### Study method and settings

This cross-sectional study was conducted among married women living in selected informal urban settlements and communities of Dhaka North and South city corporations and Gazipur city corporation, where the urban health and demographic surveillance system (UHDSS) was implemented. The baseline information on the pregnant women was gathered from the urban health and demographic surveillance system (UHDSS), one of the biggest surveillance systems of informal urban settlements in Bangladesh. The surveillance system has been running since 2015, directed by the health system and population studies division of the International Centre for Diarrhoeal Disease Research, Bangladesh (icddr,b). The total five most significant informal settlements -i.e., Mirpur, Korail (Dhaka North), Shaympur, Dhalpur (Dhaka South), and Tongi (Gazipur) were under the surveillance system. The field workers were recruited and trained to gather information quarterly on basic socio-demographic characteristics and events, including pregnancy outcomes (live births, stillbirths, induced and spontaneous abortions) for surveillance. The details of the surveillance were reported elsewhere [37].

We included all married women (age < 49 years) with pregnancy outcomes from July 2020 to December 2021. The baseline information on these pregnant women was gathered from the UHDSS database. Among those, we identified the women who had experienced any form of abortion during the study period and found 361 spontaneous abortions and 169 induced abortions. We consider one and a half years based on the Broen et al. (2005) study, which mentions the adverse effects of pregnancy on women’s mental health over time. Thereby, we conducted a cross-sectional survey among those women with spontaneous abortions. At the time of the survey, 115 women had migrated from the surveillance areas, since this population is also known as the floating population because of their remarkably high migration rate, which is a result of unstable living conditions and nature of jobs [38]. Furthermore, the field team approached the rest of the 246 women who met the inclusion criteria of having a spontaneous abortion. The final sample size included 240 who agreed to participate (Six declined to participate in the study; response rate: 97.56%).

### Data collection process

A semi-structured Bangla (native language) questionnaire (since this was a quantitative study) was developed through an extensive literature review (supplementary file 1) and was later developed into the Java-SQLite (a Structured Query Language based) software. This digital questionnaire was mounted on Android tablets to be administered by trained UHDSS field workers. These field workers were also trained by the research team to collect sensitive mental health, reproductive healthcare service utilization and abortion-related information from the vulnerable women.

The field workers were given lists containing the names and household information of 246 participants extracted from UHDSS data. They tracked the respective participants and approached them for an interview time and place convenient to them. Proper privacy was maintained during the interview by conducting it in the absence of the husbands and other members of the family.

All the interviews were conducted at the participants’ households as per their preference. The questionnaire had sections on socio-demographic characteristics collected from the UHDSS database, sexual and reproductive health-related (SRHR) knowledge, receiving post-abortion care (PAC), and experience of violence. Moreover, the assessment of anxiety and depression was done using two psychometric assessment tools: the General Anxiety Disorder (GAD-7) and the Patient Health Questionnaire (PHQ-9) [15, 39].

### Variables of interest

#### Dependent variables

The scores of Generalized Anxiety Disorder (GAD-7) and Patient Health Questionnaire (PHQ-9) scales were considered as the dependent variables for this study.

#### Generalized anxiety disorder scale (GAD-7)

Anxiety symptoms among the participants were assessed by the Generalized Anxiety Disorder Scale (GAD-7) [34]. The GAD-7 scale consists of seven items, and this scale has been validated and used in Bangladesh in several studies [40, 41]. Participants were asked how often they had experienced each symptom during the last two weeks. The response was scored on a four-point Likert scale (0 = not at all, 1 = several days, 2 = more than half the days, and 3 = nearly every day) [34]. The total scale ranges from 0 to 21. The symptom severity scores were 0–4 for minimal, 5–9 for mild, 10–14 for moderate, and 15–21 for severe [23, 35]. For the purpose of modeling, GAD-7 was considered into two categories: minimal renamed “no anxiety”, and others three groups, “presence of anxiety”. In this study, the case of anxiety was considered when women had the mild to severe anxiety category [2–4]. The internal consistency of the GAD-7 is reported as excellent (Cronbach = 0.8136) with an average inter-item covariance of 0.477.

#### Patient health questionnaire (PHQ-9)

Patient Health Questionnaire (PHQ-9), which has previously been validated in Bangladesh, was used to evaluate the level of depressive symptoms [42]. It has nine items, each with a four-point Likert scale ranging from 1 to 4 (0 = not at all, 1 = several days, 2 = more than half the days, and 3 = nearly every day). The total score ranges from 0 to 27. The scores for symptom severity were counted as 0–4 for minimal, 5–9 for mild, 10–14 for moderate, 15–19 for moderately severe, and 20–27 for severe symptoms of depression [43]. Additionally, for the model, PHQ-9 was categorized into two groups, i.e., no depressive symptoms and the presence of depressive symptoms. The minimal category was considered as no depressive symptoms, and the rest of the categories were considered to have depressive symptoms. The case of depression was considered when women’s scores in the PHQ-9 included them in the mild to severe depression category [2–5]. The Cronbach’s alpha of the PHQ-9 in the present study was estimated at 0.8628, with average inter-item covariance of 0.433.

#### Independent variables

Socio-demographic variables included age and year of education as continuous variables, household wealth index (poorer, poor, and wealthier), and working status (unemployed and employed). In addition, the history of live birth (no live birth and live birth), stillbirth (no stillbirth and stillbirth), and contraceptive use (no method and any contraceptive method) were also included. The principal component analysis was used to calculate the factor score of each variable, and the index was constructed as a weighted sum of these items. The index scores were ordered ascendingly and classified into three categories matching the economic status of the slum population. The women were classified as poorer if they belonged to ≤ 20th quintile, poor if they belonged to the 21st to 79^th,^ and wealthier if more than the 80th quintile. Having SRHR knowledge, receiving post-abortion care (PAC) (yes, no), and experiencing any violence, either physical or verbal (yes, no), were also considered as explanatory variables for the mental health status of the women.

### Statistical analysis

Descriptive analysis (percentage distribution, mean, and standard error), bivariate and logistic regression analyses were performed to assess the correlates of depressive and anxiety symptoms. Heteroscedasticity was tested using the Breuch-Pagan test for heteroscedasticity, and multicollinearity was tested using the variance influence factor (VIF) at the accepted score 5.00 [44]. A *p*-value less than 0.05 was considered significant with a 95% CI. All statistical analyses were performed in the STATA windows version 15.0 (Stata. Corp, TX).

## Results

### Characteristics of the participants

The mean age of all participants was 26.12 (± 0.394) years, whereas the mean age of women with depressive symptoms and anxiety was 26.27 (± 0.457) years and 26.52 (± 0.523) years respectively. Participants had, a 4.86 (± 0.242) years of schooling and were mostly unemployed in both of the mental health outcomes in general. More than half of the women (56.7%) lived in poor households, followed by the wealthier (22.08%) and poorer (21.25%) quintiles. Approximately, one-third (33%) of the women had no live births, used contraceptives, and 12% of the respondants had previous experience of stillbirths at the time of the survey (Table 1). The majority (77.50%) of women developed depressive symptoms, whereas more than half (58.75%) of the respondents reported to have anxiety following a spontaneous abortion. The distribution of socio-economic and reproductive health-related attributes of the women with depressive symptoms and anxiety is given in Table 1.

**Table 1:** Percentage distribution of the socio-demographic characteristics and reproductive history of the women experienced spontaneous abortion

Variables	Prevalence of GAD (*N* = 141, 58.75%)	Prevalence of PHQ (*N* = 186, 77.50%)	Total (*N* = 240)
***Age***	26.52 (0.523)	26.27 (0.457)	26.12 (0.394)
***Education***	4.55 (0.294)	4.80 (0.274)	4.86 (0.242)
***Working status***			
Unemployed	97 (68.79%)	135 (72.58%)	168 (70.00%)
Employed	44 (31.21%)	51 (27.42%)	72 (30.00%)
***Wealth index***			
Poorer	36 (25.53%)	41 (22.04%)	51 (21.25%)
Poor	76 (53.90%)	103 (55.38%)	136 (56.67%)
Wealthier	29 (20.57%)	42 (22.58%)	53 (22.08%)
***Live birth***			
No live birth	46 (32.62%)	60 (32.26%)	79 (32.92%)
Live birth	95 (67.38%)	126 (67.74%)	161 (67.08%)
***Stillbirth***			
No stillbirth	125 (88.65%)	161 (86.56%)	211 (87.92%)
Stillbirth	16 (11.35%)	25 (13.44%)	29 (12.08%)
***Use contraception***			
No	92 (65.25%)	127 (68.28%)	159 (66.25%)
Yes	49 (34.75%)	59 (31.72%)	81 33.75%)

### Prevalence of depressive symptoms and anxiety

**Fig. 1:**
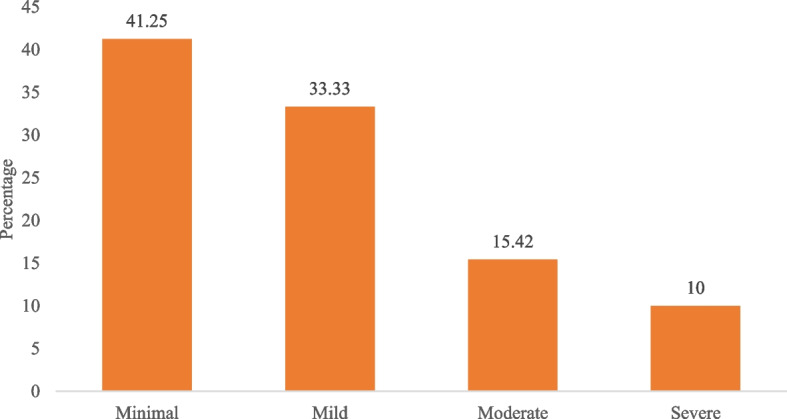
Prevalence of anxiety among the slum women with experience of spontaneous abortion

**Fig. 2:**
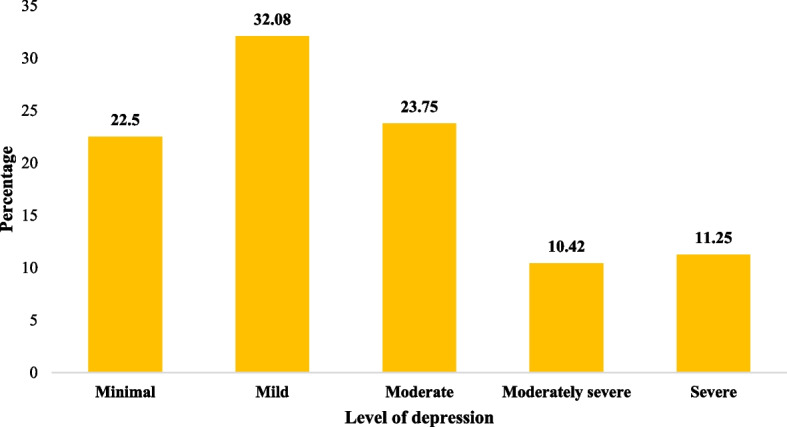
Prevalence of depression among the slum women with experience of spontaneous abortion

One of every ten women had severe forms of depressive symptoms and anxiety (moderately severe to severe, 10.40–11.30%). The prevalence of women with moderate levels of depressive symptoms was higher than that of women with moderate levels of anxiety (23.80% vs. 15.40%). One in three women had mild levels of depressive symptoms (32.10%) and anxiety (33.30%) (Figs. 1 and 2). Additionally, more women experienced depressive symptoms than anxiety after the spontaneous termination of the pregnancy.

### Associated factors of depressive symptoms and anxiety

Based on logistic regression, education, SRHR knowledge, and receiving PAC were significantly associated with higher GAD scores (Table 2). On the other hand, working status, SRHR knowledge, and taking PAC were significantly associated with PHQ scores (Table 3). With every increasing unit of classes of formal education, there was a 18% reduction in the odds of developing anxiety (Adjusted Odds Ratio- AOR: 0.82; 95% CI: 0.68–0.98). Women who received post-abortion care (PAC) had a 90% lower risk of developing anxiety (AOR: 0.10; 95% CI: 0.020–0.544) and depressive symptoms (AOR: 0.10; 95% CI: 0.012–0.893) compared to those who did not. On the contrary, the chance of developing depressive symptoms and anxiety was more than 70% higher for the women who has higher knowledge about their SRHR (AOR: 1.72; 95% CI: 1.248–2.358) and depression (AOR: 1.71; 95% CI: 1.125–2.610). Overall models were statistically significant for anxiety (F (8,108) = 2.85, *p* < 0.05) and depressive symptoms (F (8,321) = 2.31, *p* < 0.05).

**Table 2:** Possible Predictors of the anxiety among the women with experience of spontaneous abortion

Factors	Anxiety (GAD score)				
	COR	95% CI	AOR	95% CI	VIF
***Age***	1.06 (0.056)	0.955, 1.178	1.02 (0.057)	0.914, 1.140	1.20
***Education***	0.87 (0.076)	0.731, 1.030	0.82 (0.076)*	0.680, 0.982	1.21
***Wealth index***					
Poorer	1.00		1.00		
Poor	0.41 (0.342)	0.080, 2.115	0.37 (0.305)	0.074, 1.873	
Wealthier	0.31 (0.308)	0.044, 2.193	0.28 (0.281)	0.039, 2.015	
***Working status***					
Unemployed	1.00		1.00		
Employed	0.60 (0.430)	0.148, 2.455	0.50 (0.363)	0.121, 2.088	1.12
***SRHR knowledge***	1.53 (0.239)**	1.127, 2.085	1.72 (0.277)**	1.248, 2.358	1.15
***Receiving PAC services***					
No	1.00		1.00		
Yes	0.17 (0.139)*	0.031, 0.870	0.10 (0,087)**	0.020, 0.544	1.03
***Experience abuse***					
No	1.00		1.00		
Yes	1.10 (0.912)	0.214, 5.636	1.20 (0.992)	0.237, 6.110	1.06

*indicates *p* < 0.05 **indicates *p* < 0.01

*COR* Crude Odds Ratio, *AOR* Adjusted Odds ratio

**Table 3:** Possible Predictors of the depressive symptoms among the women with experience of spontaneous abortion

Summary statistics	
Observation	240
F(8,108)	2.85
*p*-value	< 0.05
R^2^	0.0899
Adj. R^2^	0.0584
RMSEs	4.9045
Breusch-pagan test for heteroscedasticity	0.26
Breusch-pagan test for heteroscedasticity (p-value)	0.6084

## Discussion

Spontaneous abortions are known to have long-lasting and severe effects on mothers. A pregnancy loss has been known to cause a lack of self-efficacy, hinder relationships with family members and decrease quality of life [45, 46]. However, the studies on these relationships were either conducted in developed countries or focused on the physiological repercussions. Therefore, the psychological consequences of loss of pregnancy in disadvantaged populations in developing countries were lacking.

To the best of our knowledge, this paper is the first to evaluate the impact of spontaneous abortion on mental health among the women slum dwellers of Bangladesh. The findings of this study can encourage policies to restructure the existing maternal health care services and improve the well-being of women who belong to the most disadvantaged portion of the population.

Our study reported a mild to severe anxiety and depressive symptoms rate of 58.75% and 77.50%, respectively, among the women who had spontaneous abortions. We included the mild category for both mental health conditions as it is regarded as the early stage, and early detection of these difficulties will better ensure the impact of any therapeutic intervention, preventing the onset of complex mental health disorders [47]. The prevalences of depressive symptoms and anxiety are much higher than the prevalences (depression: 7%, anxiety: 4.20%) reported in the National Mental Health Survey-2018 of Bangladesh [48]. Moreover, the rate of depressive symptoms in women who experienced spontaneous abortions in our study is higher than that of Sri Lanka, and the level of anxiety is higher than that of Eastern and Southern African countries [49, 50]. So, it is crucial to address this greater burden of mental health consequences among slum women, as this can also affect their subsequent pregnancy and compromise the overall fertility span [45, 51].

Our findings stated that women from urban slums who received a higher level of education had lower anxiety symptoms. It has been reported that low education in women increases the possibility of accepting misinformation, overestimating risks, and underestimating the benefits of abortion services [52, 53]. These misconceptions about the health risks of abortion may exacerbate anxiety among women with lower education about the procedure and affect their ability to cope afterwards [52–56]. In addition, such misperceptions generate negative stereotypes about women who have abortions, contributing to stigma and exaggerating the risks of the abortion procedure [55–57]. On the other hand, women with more education are expected to be aware of the safety and consequences of abortion and contraception, making them more resilient and denoting less restrictive abortion beliefs [52]. Moreover, evidence supports that integrating self-care education into prenatal service programs effectively reduces anxiety and depression after a spontaneous abortion [41]. Therefore, ensuring education for women living in slums that incorporates SRHR and mental health components would be crucial to reducing the consequences of an abortion.

In addition, a lower depression score through PHQ in slum women was found in those who were employed compared to unemployed women. Past studies also reported that working status could improve physical and mental health outcomes, especially lowering depression among individuals [58]. Financial stability gives women the autonomy to make decisions and enables them to seek healthcare quickly [59]. Moreover, peer support in the workplace increases the opportunity to share inner distress, learn about abortion-related information, and get support from women with similar experiences, which can help reduce mental health complications [60, 61]. The low affordability of quality PAC services has been linked to depressive symptoms in slum women [62]. Therefore, promoting women’s participation in economic work is crucial, enabling them to exercise the decision-making process and increase their access to need-based healthcare services. [51].

The multivariate analysis reported that increased levels of anxiety and depressive symptoms were associated with having higher SRHR knowledge. Global studies support that the prevalence of spontaneous abortions is independent of the woman’s will or even her understanding [63]. Our study findings reported significantly higher scores of GAD-7 and PHQ-9 among the women with a higher level of SRHR knowledge. This might be due to their inability to prevent the event despite having SRHR knowledge. Furthermore, a higher understanding of SRHR does not ensure uptake and compliance with the services by women [63, 64]. Hence, the community-based promotion of SRHR services might increase women’s reproductive healthcare-seeking behavior and utilization of services which will aid in limiting psychological complications [65].

Our study also reported that receiving post-abortion care (PAC) was significantly associated with lower rates of anxiety and depressive symptoms. As per the evidence, delaying or not receiving post-abortion care might cause inescapable losses, such as physical disability and losing childbearing capacity, leading to further mental health complications [66]. Standard post-abortion care usually comprises quality screening and counseling procedures which can help minimize psychological impacts among women [65, 67]. In Bangladesh, formal PAC care services only include support for incomplete abortions and advice regarding family planning with no psychosocial support services [68]. However, evidence supports psychosocial support services combined with appropriate medical consultation can reduce grief and improve women’s functioning through proper coping guidance and information about future pregnancies [69, 70]. Therefore, further research could explore integrating psychological counseling by training service providers to provide basic psychosocial support and follow-up care in PAC. These could be possible solutions to mitigate the psychological burden among the women who experienced pregnancy loss [69]. In addition, increasing awareness about the availability and importance of accessing PAC services should be ensured to improve the quality of life of the slum women who have had a miscarriage or induced abortion.

### Implications for future practice

The findings highlight important considerations for public health policy and the provision of mental health and SRH services for socially disadvantaged women, particularly those who live in urban slums. Recent studies highlight the importance of fulfilling SRHR needs to improve the overall well-being of women. Since poorer mental health outcomes occur among women after a spontaneous abortion, it is critical to enhance existing PAC services by including mental health services such as post-abortion mental health counseling, psychiatric medications, follow-up, and referral for severe psychological risks. Also, it is essential to investigate the women’s experiences and healthcare needs following abortion. Moreover, it is necessary to raise community-level awareness, reduce stigma and build capacity of local community-level healthcare workers to provide basic psychosocial support, and SRH care for low-income women and adolescents.

### Strengths and Limitations

A few limitations should be considered while interpreting the findings. One of the potential limitations was that, as a cross-sectional study, it did not establish any cause-and-effect relationships among variables. Future studies assessing anxiety and depressive symptoms among women who did not experience a spontaneous abortion should be conducted to better understand the situation. Moreover, abortion-related data was collected via a retrospective inquiry, which may lead to recall bias and memory lapse. In line with this, the current study explored mental health soon after the event, but the long-term effects and possible demographic predictors should also be investigated. Additionally, this study only included quantitative data; and future studies should be conducted to explore in-depth women’s experiences after an abortion. Lastly, the study was conducted on women living in the selected informal settelments of Dhaka city, and the findings are not generalizable to all slums in Bangladesh.

Despite the limitations, one of the major strengths of this study was that, to the authors’ best knowledge, it is one of the first in Bangladesh to explore the mental health conditions of women with a history of abortion. Additionally, it assessed it in a population of marginalized women largely understudied in research. Moreover, the data were collected from a surveillance system, which followed a scientific approach for tracking the demographic and health outcomes of the slum population, which could be beneficial for designing large-scale studies. Furthermore, as the population is under a surveillance system, monitoring and evaluation of the behavioral change is possible in the future if an intervention is provided by comparing the data.

## Conclusion

The present study is one of the first, to the authors’ knowledge ever, to explore the association between spontaneous abortions and the mental health conditions of slum dwellers in Dhaka, Bangladesh. It provides evidence that the rates of anxiety and depressive symptoms amongst this vulnerable population are very high and provided an indication as to which factors could be driving them. The findings showed that higher education and employment rates among women are protective against mental health difficulties after an abortion; this highlights the need to ensure that slum dwellers have access to education. In addition, this study also recommends empowering women by improving their participation in economic sectors, paving the way for increased autonomy and decision-making rights. Most importantly, this study showed that accessing PAC services helped prevent mental health distress, and therefore, immediate action is required to integrate mental healthcare into the formal PAC service package while ensuring its availability, accessibility, and affordability in Bangladesh.

## Supplementary Information

**Additional file 1.**

## Data Availability

The datasets generated during and/or analyzed during the current study are available from the author on reasonable request.

## Abbreviations

AOR: Adjusted Odds Ration
GAD-7: General Anxiety Disorder-7
PHQ-9: Patient Health Questionnaire-9
PAC: Post-abortion care
SRH: Sexual and reproductive health
SRHR: Sexual and reproductive health rights
UHDSS: Urban Health and Demographic Surveillance System
WHO: World Health Organization
