# Supplementary material for: Depressive symptoms and anxiety among women with a history of abortion living in urban slums of Bangladesh
Source: BMC Psychol. 2023 Jul 4;11:197. doi: 10.1186/s40359-023-01224-0 (PMC10318742; doi:10.1186/s40359-023-01224-0)
Supplement: Supplementary file 1 — Additional file 1. [file 40359_2023_1224_MOESM1_ESM.docx]

Questionnaire

Gaps in perception in SRHR, barriers to safe MR and post abortion care services and related gender- based violence in poor young women in Dhaka slums: A comprehensive study

Date of interview: ……../………/………

| Sl | Questions | Response | Coding | Skip note | Remarks |
| --- | --- | --- | --- | --- | --- |
| Socio-Demographics | | | | | |
|  | HH ID |  |  |  |  |
|  | Respondent’s Name |  |  |  |  |
|  | Respondent’s ID |  |  |  |  |
|  | Respondent’s age (from record) | …….year |  |  |  |
|  | Marital Status (from record) |  |  |  |  |
|  | Slum:  Korail  Mirpur  Shyampur  Dhalpur  Tongi  Ershadnagar |  | (Code from record) |  |  |
|  | Education (From Record) |  |  |  |  |
|  | Occupation (From record) |  |  |  |  |
| Reproductive Health | | | | | |
|  | Children | No children | 0 |  |  |
|  |  | Have children…….(number) |  |  |  |
|  | How long you have been married for? | ………year |  |  |  |
|  | Age during marriage | ……… year |  |  |  |
|  | Age of partner during marriage | ……… year |  |  |  |
|  | Did you ever talk with your husband/partner about contraception? | No | 0 |  |  |
|  |  | Yes | 1 |  |  |
|  | Are you currently using any contraceptive method? | No | 0 | Skip others in the list if response is ‘0’ |  |
|  |  | Condom | 1 |  |  |
|  |  | Pill | 2 |  |  |
|  |  | Injection | 3 |  |  |
|  |  | IUD | 4 |  |  |
|  |  | Safe period | 5 |  |  |
|  |  | Withdrawal | 6 |  |  |
|  |  | Other | 999 |  |  |
| Knowledge on SRHR (MR PAC) | | | | | |
|  | Have you heard about menstrual regulation and post abortion care? If yes, from which source? | No |  | Skip following, if response is ‘no’ |  |
|  |  | Parents |  |  |  |
|  |  | Husband |  |  |  |
|  |  | Other family members |  |  |  |
|  |  | Friends |  |  |  |
|  |  | Mass media |  |  | TV, Newspaper, radio etc |
|  |  | Social media |  |  | Facebook, instgram, etc |
|  |  | Others (specify) |  |  |  |
|  | A woman can get pregnant on the very first time that she has sexual intercourse | False | 0 |  |  |
|  |  | True | 1 |  |  |
|  |  | Don’t Know | 99 |  |  |
|  | A woman stops growing after she has had sexual intercourse for the first time. | False | 0 |  |  |
|  |  | True | 1 |  |  |
|  |  | Don’t Know | 99 |  |  |
|  | A woman can get pregnant if she has sexual intercourse during her periods. | False | 0 |  |  |
|  |  | True | 1 |  |  |
|  |  | Don’t Know | 99 |  |  |
|  | Condoms are an effective method of preventing pregnancy | False | 0 |  |  |
|  |  | True | 1 |  |  |
|  |  | Don’t Know | 99 |  |  |
|  | Condom Reduce risk of STIs and HIV/AIDS | False | 0 |  |  |
|  |  | True | 1 |  |  |
|  |  | Don’t Know | 99 |  |  |
|  | Emergency pills should be taken within 72 hours of unprotected sex | False | 0 |  |  |
|  |  | True | 1 |  |  |
|  |  | Don’t Know | 99 |  |  |
|  | What is the optimum age of initiation of sexual intercourse? | ………….. years |  |  |  |
|  | STDs transmit through unprotected sex | False | 0 |  |  |
|  |  | True | 1 |  |  |
|  |  | Don’t Know | 99 |  |  |
|  | Abortion is unsafe when performed without medical supervision. | False | 0 |  |  |
|  |  | True | 1 |  |  |
|  |  | Don’t Know | 99 |  |  |
|  | What complications may arise from unsafe abortion? | Bleeding/Hemorrhage | 1 |  |  |
|  |  | Infection/sepsis | 2 |  |  |
|  |  | Shock | 3 |  | *Faint caused by excessive bleeding and infection/sepsis* |
|  |  | Incomplete abortion | 4 |  |  |
|  |  | Death | 5 |  |  |
|  |  | infertility | 6 |  |  |
|  |  | Don’t Know | 99 |  |  |
| History of MR, PAC | | | | | |
|  | How many pregnancy losses (stillbirths, miscarriages, abortions) have you had (including the current one)? | ………… |  |  |  |
|  | How many induced abortions have you had? | ………… |  |  |  |
|  | Why did you abort the conception last time? | It was unwanted/unplanned | 1 |  |  |
|  |  | Social issues | 2 |  |  |
|  |  | Financial issues | 3 |  |  |
|  |  | Other | 999 |  |  |
|  | Whom did you seek abortion care? | Self | 1 |  | Skip Q-… if response is ‘1’ |
|  |  | Traditional healers |  |  |  |
|  |  | Local pharmacy |  |  |  |
|  |  | MR facility |  |  |  |
|  |  | Medical Doctor |  |  |  |
|  |  | Other |  |  |  |
|  | What treatment/care they offered? | Medicine | 1 |  |  |
|  |  | Menstrual Regulation | 2 |  |  |
|  |  | Others | 3 |  |  |
|  | Have you had any complications with the induced abortion you underwent last time? | No | 0 |  |  |
|  |  | Pain |  |  |  |
|  |  | Excessive bleeding |  |  |  |
|  |  | Fever/infection |  |  |  |
|  |  | Shock |  |  |  |
|  |  | Incomplete abortion |  |  |  |
|  | Did you receive post abortion care? | No |  | Skip Q-31, if response is ‘0’ |  |
|  |  | Yes |  |  |  |
|  | Where did you receive Post abortion care from? | NGO health care providers | 1 |  |  |
|  |  | Public facilities | 2 |  |  |
|  |  | Neighbour’s advice | 3 |  |  |
|  | Did you receive post abortion family planning methods? If yes, what was it? | No | 0 | Skip the following if response is ‘0’ |  |
|  |  | Pill | 1 |  |  |
|  |  | IUD | 2 |  |  |
|  |  | Injectable method | 3 |  |  |
|  |  | Condom | 4 |  |  |
|  |  | others | 99 |  |  |
| Barriers to access MR, PAC services | | | | | |
|  | What were the top 5 barriers for you in accessing MR, PAC services? Please specify according to priority. | Family didn’t allow |  |  | *Put code according to priority* |
|  |  | Social stigma |  |  |  |
|  |  | Unaffordable costs |  |  |  |
|  |  | Didn’t know from where to access service |  |  |  |
|  |  | Distanced location of service center |  |  |  |
|  |  | Didn’t know any |  |  |  |
|  |  | Others (specify) |  |  |  |
| Impact of Covid on accessing service | | | | | |
|  | What were the top 3 difficulties you faced in accessing MR, PAC services during COVID-19? Please specify according to priority. | Economic hardship |  |  | *Put code according to priority* |
|  |  | Closure of service |  |  |  |
|  |  | Fear of transmission of infection |  |  |  |
|  |  | Didn’t know any |  |  |  |
|  |  | Others (specify) |  |  |  |
|  | | | | | |
|  | Did you get family planning counselling before abortion/MR? | No |  |  | *Put code according to priority* |
|  |  | Yes |  |  |  |
|  | Did you receive abortion/MR services despite of your unwillingness: | No |  |  | *Put code according to priority* |
|  |  | Yes |  |  |  |
|  | Did you experience any kind of emotional or verbal abuse from an immediate family member (husband, mother in law or others) after receiving abortion/MR services? | No |  |  | *Put code according to priority* |
|  |  | Yes |  |  |  |

DEPRESSION SCALE: (PHQ-9)

| Questions | Not at all | Several days | More than half the days | Nearly every day |
| --- | --- | --- | --- | --- |
| 1. Little interest or pleasure in doing things? |  |  |  |  |
| 2. Feeling down, depressed, or hopeless? |  |  |  |  |
| 3. Trouble falling or staying asleep, or sleeping too much? |  |  |  |  |
| 4. Feeling tired or having little energy? |  |  |  |  |
| 5. Poor appetite or overeating? |  |  |  |  |
| 6. Feeling bad about yourself - or that you are a failure or have let yourself or your family down? |  |  |  |  |
| 7. Trouble concentrating on things, such as reading the newspaper or watching television? |  |  |  |  |
| 8. Moving or speaking so slowly that other people could have noticed? Or the opposite - being so fidgety or restless that you have been moving around a lot more than usual? |  |  |  |  |
| 9. Thoughts that you would be better off dead, or of hurting yourself in some way? |  |  |  |  |

ANXIETY SCALE: (GAD-7)

| Questions | Not at all | Several days | More than half the days | Nearly every day |
| --- | --- | --- | --- | --- |
| 1. Feeling nervous, anxious, or on edge |  |  |  |  |
| 2. Not being able to stop or control worrying |  |  |  |  |
| 3. Worrying too much about different things |  |  |  |  |
| 4. Trouble relaxing |  |  |  |  |
| 5. Being so restless that it's hard to sit still |  |  |  |  |
| 6. Becoming easily annoyed or irritable |  |  |  |  |
| 7. Feeling afraid as if something awful might happen |  |  |  |  |
